# Toxoplasma gondii infection associated with inflammasome activation and neuronal injury

**DOI:** 10.1038/s41598-024-55887-9

**Published:** 2024-03-04

**Authors:** Dimitrios Andreou, Nils Eiel Steen, Lynn Mørch-Johnsen, Kjetil Nordbø Jørgensen, Laura A. Wortinger, Claudia Barth, Attila Szabo, Kevin S. O’Connell, Tove Lekva, Gabriela Hjell, Ingrid Torp Johansen, Monica B. E. G. Ormerod, Unn K. Haukvik, Pål Aukrust, Srdjan Djurovic, Robert H. Yolken, Ole A. Andreassen, Thor Ueland, Ingrid Agartz

**Affiliations:** 1 Department of Psychiatric Research, Diakonhjemmet Hospital, Forskningsveien 7, 0373 Oslo, Norway; 2 Norwegian Centre for Mental Disorders Research (NORMENT), Institute of Clinical Medicine, University of Oslo, Oslo, Norway; 3 Centre for Psychiatry Research, Department of Clinical Neuroscience, Karolinska Institutet and Stockholm Health Care Services, Stockholm County Council, Stockholm, Sweden; 4 Norwegian Centre for Mental Disorders Research (NORMENT), Division of Mental Health and Addiction, Oslo University Hospital, Oslo, Norway; 5 Department of Psychiatry and Department of Clinical Research, Østfold Hospital, Grålum, Norway; 6 Division of Mental Health and Addiction, Vestre Viken Hospital Trust, Drammen, Norway; 7 Department of Medical Genetics, Oslo University Hospital, Oslo, Norway; 8 Research Institute of Internal Medicine, Oslo University Hospital, Rikshospitalet, Oslo, Norway; 9 Department of Forensic Research and Education, Oslo University Hospital, Oslo, Norway; 10 Institute of Clinical Medicine, University of Oslo, Oslo, Norway; 11 Section of Clinical Immunology and Infectious Diseases, Oslo University Hospital, Rikshospitalet, Oslo, Norway; 12 Norwegian Centre for Mental Disorders Research (NORMENT), Department of Clinical Science, University of Bergen, Bergen, Norway; 13 Stanley Division of Developmental Neurovirology, Department of Pediatrics, Johns Hopkins University School of Medicine, Baltimore, MD USA; 14 Thrombosis Research Center (TREC), Division of Internal Medicine, University Hospital of North Norway, Tromsø, Norway

**Keywords:** Neuron-specific enolase, Interleukin-18, Schizophrenia, Bipolar disorder, Healthy controls, Infection, Bipolar disorder, Schizophrenia

## Abstract

Toxoplasma gondii (TOXO) infection typically results in chronic latency due to its ability to form cysts in the brain and other organs. Latent toxoplasmosis could promote innate immune responses and impact brain function. A large body of evidence has linked TOXO infection to severe mental illness (SMI). We hypothesized that TOXO immunoglobulin G (IgG) seropositivity, reflecting previous infection and current latency, is associated with increased circulating neuron-specific enolase (NSE), a marker of brain damage, and interleukin-18 (IL-18), an innate immune marker, mainly in SMI. We included 735 patients with SMI (schizophrenia or bipolar spectrum) (mean age 32 years, 47% women), and 518 healthy controls (HC) (mean age 33 years, 43% women). TOXO IgG, expressed as seropositivity/seronegativity, NSE and IL-18 were measured with immunoassays. We searched for main and interaction effects of TOXO, patient/control status and sex on NSE and IL-18. In the whole sample as well as among patients and HC separately, IL-18 and NSE concentrations were positively correlated (p < 0.001). TOXO seropositive participants had significantly higher NSE (3713 vs. 2200 pg/ml, p < 0.001) and IL-18 levels (1068 vs. 674 pg/ml, p < 0.001) than seronegative participants, and evaluation within patients and HC separately showed similar results. Post-hoc analysis on cytomegalovirus and herpes simplex virus 1 IgG status showed no associations with NSE or IL-18 which may suggest TOXO specificity. These results may indicate ongoing inflammasome activation and neuronal injury in people with TOXO infections unrelated to diagnosis.

## Introduction

Toxoplasma gondii (TOXO) is an intracellular parasite with a high seroprevalence affecting 30–65% of the human population worldwide^1^. Human hosts contract the infection predominantly via ingestion of oocysts from environments contaminated with cat feces or ingestion of tissue cysts in undercooked meat, while another route of infection is the transplacental^1,2^. The TOXO primary infection of immunocompetent individuals is typically either asymptomatic or oligosymptomatic with lymphadenopathy and flu-like symptoms, whereas immunocompromised and congenitally infected hosts may develop severe disease including central nervous system (CNS) pathology^2,3^. Even when infecting immunocompetent hosts, TOXO typically establishes an inapparent and life-long latent infection due to its ability to form latent tissue cysts mostly in the brain, eyes, myocardium and skeletal muscles^1^. This latent infection can be complicated with symptomatic reactivations mainly in immunodeficient hosts^3^.

Schizophrenia (SZ) and bipolar disorder (BP) comprise severe mental illnesses (SMI) with multipart etiology where both genetic predisposition and environmental exposures are implicated^4,5^. SMI are linked to increased all-cause mortality^6^ and increased risk for suicide and self-perceived incapacity^7^. A large body of evidence has linked SZ and BP to TOXO infections^8–10^. However, the mechanisms underlying these associations are largely unknown.

The host innate immune system is the first line of defense against infections. It responds to pathogens, such as TOXO, by recognizing pathogen-associated molecular patterns and inducing among others activation of the Nod-like receptor (NLR) family pyrin domain containing 3 (NLRP3) inflammasomes, which results in the release of the highly pro-inflammatory cytokines interleukin-18 (IL-18) and IL-1β^11^. Animal studies have shown that TOXO infection results in inflammasome activation and IL-18 release^12^. Furthermore, we have recently demonstrated that patients with SMI are characterized by increased IL-18 levels^13^.

Neuron-specific enolase (NSE) is the main enolase isoform expressed in the mature cells of the CNS, constituting a significant fraction of the total soluble protein of the brain^14^. As it is not actively secreted, elevated cerebrospinal and circulating levels indicate neuronal damage^15,16^. NSE is thought to be the main biomarker of neuronal damage after traumatic brain injury^17^, and is also elevated in some neurodegenerative disorders^16^. Furthermore, an animal study has shown substantially increased brain expression of NSE after TOXO infection^18^.

Based on the role of toxoplasmosis in promoting innate immune responses^19^ and impairing CNS function^20^, we hypothesized that TOXO immunoglobulin G (IgG) seropositivity, reflecting past TOXO infection and current latency, would be linked to progressive neuronal damage during the latency period and innate immune responses in an effort to contain the infection. As markers of CNS damage and innate immune activation, we evaluated NSE and IL-18 levels. We further hypothesized that putative associations between TOXO IgG seropositivity and increased NSE and/or IL-18 would be stronger in patients with SMI relative to HC.

## Material and methods

### Participants

The Thematically Organized Psychosis (TOP) research study is the main protocol for adults at the Norwegian Centre for Mental Disorders Research (NORMENT, Oslo, Norway; www.med.uio.no/norment/english). We selected here participants with available TOXO IgG, NSE and IL-18 data (from 2003 to 2017). Patients were recruited from outpatient and inpatient psychiatric units in the Oslo region, Norway while HC were recruited from the same catchment area using the national population register. Patients were evaluated with the Structured Clinical Interview (SCID-I) for the Diagnostic and Statistical Manual of Mental Disorders, fourth edition (DSM-IV)^21^; patients with SZ spectrum or BP spectrum disorders were included. HC were screened for SMI with the Primary Care Evaluation of Mental Disorders (Prime-MD)^22^; HC with previous or current psychiatric disorders including substance/alcohol use disorder or with first-degree relatives with SMI were excluded. Patients and HC with previous moderate or severe head injury, neurological disorders or medical conditions that could affect brain function were excluded.

The final sample consisted of 1253 participants: 735 patients with SMI (491 patients with SZ spectrum disorders, i.e. SZ (n = 280), schizophreniform disorder (n = 28), schizoaffective disorder (n = 73), delusional disorder (n = 34), brief psychotic disorder (n = 5) and psychotic disorder not otherwise specified (n = 71), and 244 patients with BP spectrum disorders, i.e. BP I (n = 165), BP II (n = 68) and BP not otherwise specified (n = 11), and 518 HC.

The authors assert that all procedures contributing to this work comply with the ethical standards of the relevant national and institutional committee on human experimentation and with the Helsinki Declaration. The study was approved by the Regional Committee for Medical Research Ethics South East Norway and the Norwegian Data Inspectorate. All participants gave written informed consent.

### Measures and medication

We used education years (continuous variable) as a proxy indicator for socioeconomic status for all participants^23^. We assessed handedness (right-handedness vs. left-handedness/ambidexterity), body mass index (BMI) and daily use of tobacco (%) as well as alcohol use with the alcohol use disorder identification test (AUDIT) and substance use with the drug use disorder identification test (DUDIT). We assessed the patients with the Positive and Negative Syndrome Scale (PANSS), the Inventory of Depressive Symptoms, clinician rated (IDS-C) and the Young Mania Rating Scale (YMRS). We defined the duration of illness (DOI) as the time passed since the first psychotic episode for patients with SZ spectrum disorders and since the first affective episode for patients with BP spectrum disorders. Finally, we obtained information concerning current medication use (antipsychotics, antiepileptics, antidepressants and lithium) by clinical interviews and hospital records. For patients on antipsychotics, we calculated the current chlorpromazine equivalent doses (CPZ) in mg/day^24^.

### Antibody, neuron-specific enolase, and interleukin-18 measurements

Serology measurements were obtained by standard procedures at the Stanley Neurovirology Laboratory (Johns Hopkins University School of Medicine, Baltimore, MD, USA). TOXO, cytomegalovirus (CMV) and herpes simplex virus 1 (HSV1) IgG antibody concentrations were measured by solid-phase immunoassays and expressed as dichotomous, seropositive/seronegative (TOXO+/TOXO−, CMV+/CMV−, HSV1+/HSV1−) measures. The amount of antibody was expressed in terms of the ratio of optical density of the test sample to that of the standard sample, and the cut-offs for seronegativity/seropositivity were based on standards run with each sample^25–28^.

Plasma levels of NSE and IL-18 were measured in duplicate by enzyme immunoassays using commercially available antibodies (R&D Systems, Minneapolis, MN, USA) in a 384-well format using a combination of a SELMA (Jena, Germany) pipetting robot and a BioTek (Winooski, VT, USA) dispenser/washer. Absorption was read at 450 nm with wavelength correction set to 540 nm using an ELISA plate reader (Bio-Rad, Hercules, CA, USA)^13,29^.

### Statistics

First, we explored the putative correlations between the two dependent variables (NSE and IL-18) in the whole sample as well as among TOXO+ HC, TOXO+ patients, TOXO− HC and TOXO− patients. Next, in the bivariate analysis of the whole sample, we searched for associations between TOXO IgG status and patient/control status, sex, age, education years, handedness, BMI, AUDIT score and DUDIT score (Table 1). The distributions of both dependent variables (NSE and IL-18) were highly positively skewed (Suppl. Figures 1 and 2), and the median is therefore considered as a better measure of central tendency than the mean. In addition, a logarithmic transformation normalized the NSE concentrations but logarithmic, square root or reciprocal transformations did not normalize the IL-18 concentrations (Suppl. Figures 1 and 2). An appropriate approach in the case of non-normally distributed data, especially when transformations fail to normalize the data, is the median regression which is a special case of the quantile regression, and permits adjustment for covariates and the use of interaction terms^30^. To investigate interaction effects, we first ran full factorial median regressions (TOXO IgG status, patient/control status and sex) on NSE and IL-18. The models were adjusted for age as well as for variables that significantly differentiated TOXO+ from TOXO− participants in the bivariate analysis. As TOXO+ and TOXO− participants did not differ in any of the analyzed variables (Table 1), and there were no interaction effects (results section), our final models were Mann–Whitney U tests (MWU) of the putative differences in NSE and IL-18 levels between TOXO+ and TOXO− participants.

**Table 1:** Group differences between toxoplasma immunoglobulin G (IgG) seronegative (TOXO−) and seropositive (TOXO+) participants in patient/control status, sex, age, education years, handedness (right-handedness vs. left-handedness/ambidexterity), body mass index (BMI), alcohol use disorder identification test (AUDIT) score and drug use disorder identification test (DUDIT) score in the whole sample.

	TOXO−		TOXO+		P value^b^
	N^a^	Mean (SD) or %	N^a^	Mean (SD) or %	
Patient/control status (% patients)	977	59.6	276	55.4	0.218
Sex (% females)	977	46.7	276	42.4	0.207
Age (years)	977	32.6 (10)	276	32.5 (10.5)	0.798
Education years	928	13.4 (2.6)	255	13.4 (2.3)	0.987
Handedness (right-handedness %)	927	88.9	255	89	0.953
BMI (kg/m^2^)	909	25.6 (4.6)	264	25.9 (4.7)	0.348
AUDIT	636	6.9 (6)	203	6.3 (5.2)	0.186
DUDIT	664	2.4 (6.1)	214	2 (5.4)	0.433

^a^Number of participants with data for each variable.

^b^Chi-square test or t-test.

Similarly, in the post-hoc analysis by diagnostic status, among patients with SMI and HC separately, we applied the same methodology as in the analysis of the whole sample. The variables used for the bivariate analysis of HC were sex, age, education years, handedness, BMI, tobacco use, AUDIT score and DUDIT score, while for the bivariate analysis of patients, sex, age, education years, handedness, BMI, tobacco use, AUDIT score, DUDIT score, DOI, medication variables, PANSS total score, YMRS score and IDS-C score (Table 2). Finally, in our CMV and HSV1 post-hoc analyses, we applied the same methodology as for the TOXO analysis in the whole sample.

**Table 2:** Group differences between toxoplasma immunoglobulin G (IgG) seronegative (TOXO−) and seropositive (TOXO+) patients with SMI (severe mental illness) in sex, age, education years, handedness (right-handedness vs. left-handedness/ambidexterity), body mass index (BMI), daily use of tobacco, alcohol use disorder identification test (AUDIT) score, drug use disorder identification test (DUDIT) score, duration of illness (DOI), the percentage of patients on psychotropic medications as well as the chlorpromazine equivalent doses (CPZ) among patients on antipsychotics, Positive and Negative Syndrome Scale (PANSS) total score, Young Mania Rating Scale (YMRS) score and Inventory of Depressive Symptoms, clinician rated (IDS-C) score are presented.

	TOXO−		TOXO+		P value^b^
	N^a^	Mean (SD) or %	N^a^	Mean (SD) or %	
Patients with SMI					
Sex (% women)	582	48.6	153	42.5	0.176
Age (years)	582	31.8 (10.5)	153	32.1 (11.7)	0.789
Education years	533	12.6 (2.6)	132	12.6 (2.3)	0.929
Handedness (right-handedness %)	532	89.1	132	87.1	0.521
BMI (kg/m^b^)	544	26.3 (5.2)	143	26.4 (5.2)	0.769
Tobacco use (%)	571	56	148	55.4	0.889
AUDIT	421	7.61 (6.9)	112	7 (6.4)	0.401
DUDIT	447	3.4 (7.2)	120	3.4 (6.9)	0.942
DOI (years)	562	9.2 (9.1)	150	8.7 (7.8)	0.498
On antipsychotics (%)	576	74.8	149	75.2	0.932
CPZ (mg/day)	426^c^	316.1 (256)	109^3^	318.8 (230.1)	0.921
On antiepileptics (%)	576	21.4	149	21.5	0.974
On lithium (%)	576	8.2	149	11.4	0.213
On antidepressants (%)	576	32.1	149	32.2	0.982
PANSS	570	57.3 (17.1)	150	54.4 (14.7)	0.052
YMRS	505	5 (5.3)	122	3.7 (4)	0.080^d^
IDS-C	439	18.7 (12.2)	102	17.2 (11.1)	0.269
Healthy controls					
Sex (% women)	395	43.8	123	42.3	0.766
Age (years)	395	33.2 (9)	123	33.1 (8.7)	0.953
Education years	395	14.4 (2.2)	123	14.2 (2.1)	0.262
Handedness (right-handedness %)	395	88.6	123	91.1	0.445
BMI (kg/m^2^)	365	24.5 (3.4)	121	25.2 (4)	0.122^d^
Tobacco use (%)	184	15.8	36	13.9	0.776
AUDIT	215	5.7 (3.3)	91	5.5 (3.1)	0.352
DUDIT	217	0.29 (1.6)	94	0.17 (0.63)	0.468

Group differences between TOXO− and TOXO+ healthy controls in sex, age, education years, handedness, BMI, tobacco use, AUDIT score and DUDIT score are also presented.

^a^Number of participants with data for each variable.

^b^Chi-square test or t-test.

^c^Patients currently on antipsychotics.

^d^Mann–Whitney U test due to unequal variances.

We conducted all the analyses with IBM SPSS Statistics 28.

## Results

### Main analysis

#### Interleukin-18 and neuron-specific enolase were positively correlated

In the whole sample, IL-18 and NSE were positively correlated, assessed with Spearman’s correlation, r_s_ = 0.328, p < 0.001. Stratifying by TOXO status and diagnostic status, IL-18 and NSE were still significantly correlated in all subgroups: TOXO+ HC, r_s_ = 0.417, p < 0.001, TOXO+ patients, r_s_ = 0.249, p = 0.002, TOXO− HC, r_s_ = 0.471, p < 0.001 and TOXO− patients, r_s_ = 0.238, p < 0.001.

#### Toxoplasma gondii seropositivity associated with higher levels of NSE and IL-18 in the whole sample

**Figure 1:**
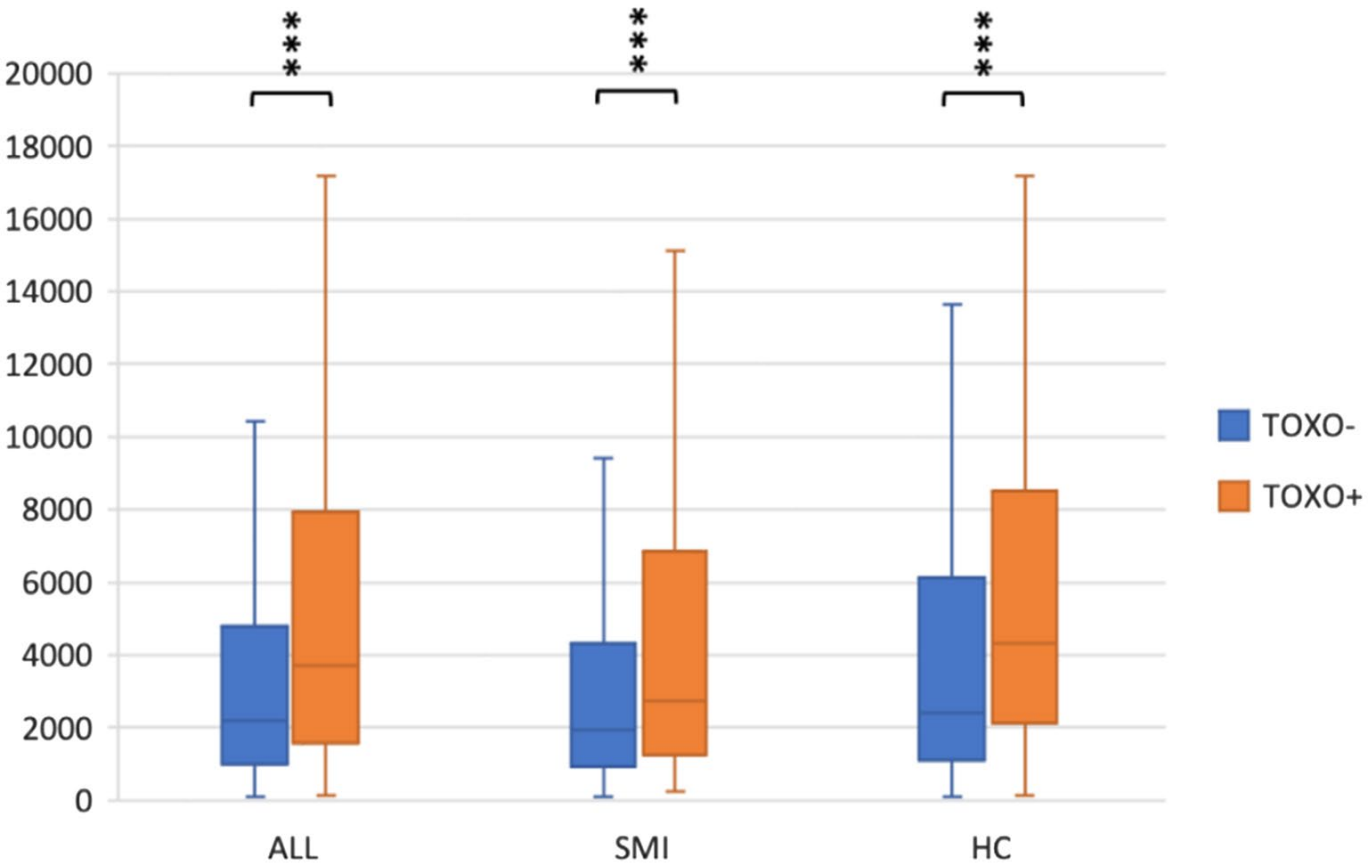
NSE in TOXO− and TOXO+ participants, patients and HC. Neuron-specific enolase (NSE) concentrations (pg/ml) in toxoplasma seronegative (TOXO−) and seropositive (TOXO+) participants (n = 1253), patients with severe mental illness (SMI) (n = 735) and healthy controls (HC) (n = 518). Among all participants, patients with SMI and HC, TOXO+ participants had significantly higher NSE than TOXO− participants, assessed with Mann–Whitney U tests. Medians (horizontal lines), interquartile ranges and whiskers with restricted length to a maximum of 1.5 times the interquartile range are shown. ***p < 0.001.

**Figure 2:**
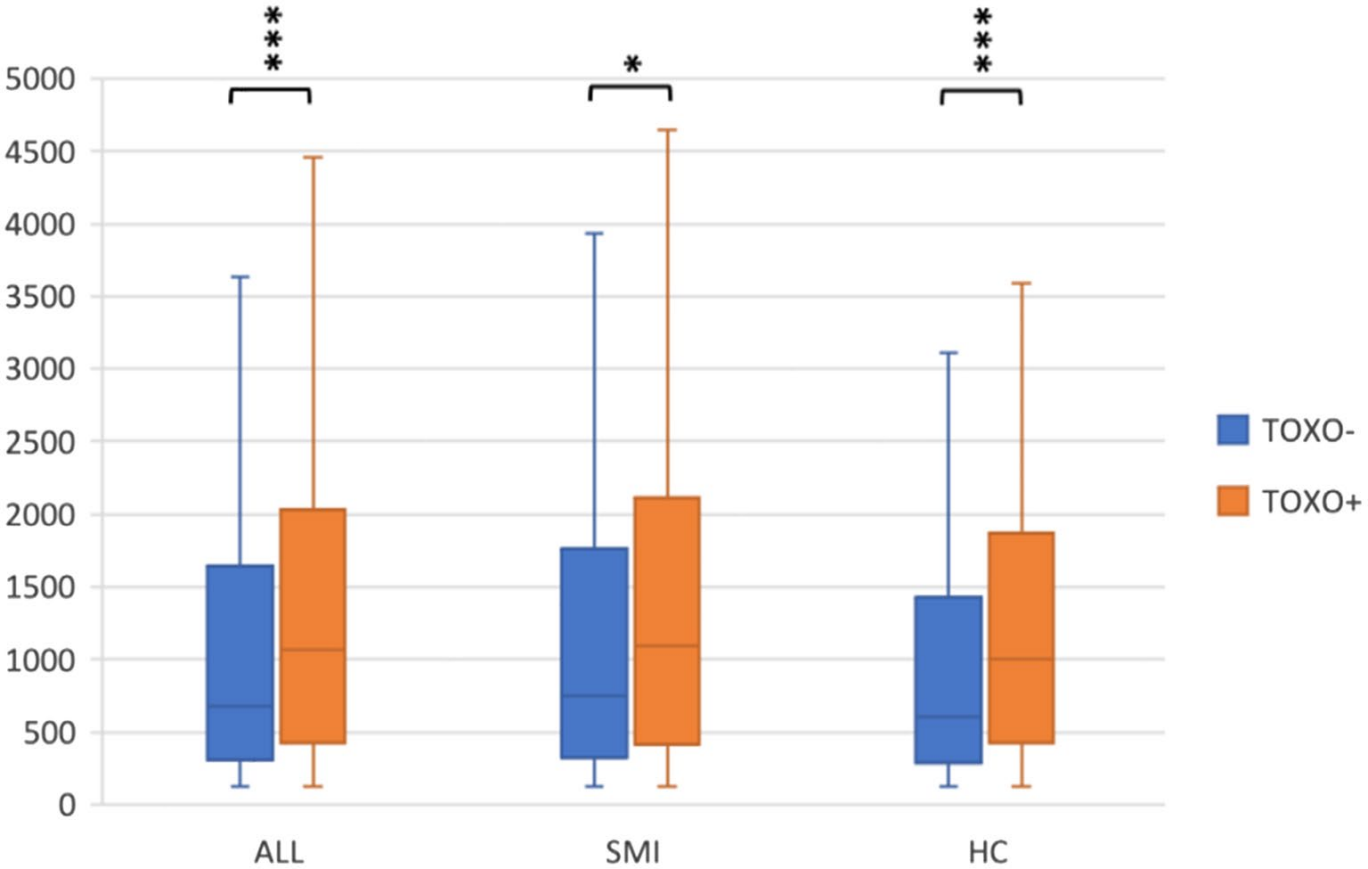
IL-18 in TOXO− and TOXO+ participants, patients and HC. Interleukin-18 (IL-18) concentrations (pg/ml) in toxoplasma seronegative (TOXO−) and seropositive (TOXO+) participants (n = 1253), patients with severe mental illness (SMI) (n = 735) and healthy controls (HC) (n = 518). Among all participants, patients with SMI and HC, TOXO+ participants had significantly higher IL-18 than TOXO− participants, assessed with Mann–Whitney U tests. Medians (horizontal lines), interquartile ranges and whiskers with restricted length to a maximum of 1.5 times the interquartile range are shown. *p = 0.015. ***p < 0.001.

In the bivariate analysis of the whole sample (n = 1253) there were no significant differences between TOXO− and TOXO+ participants in diagnostic group status (patient/control), sex, age, education years, handedness, BMI, AUDIT or DUDIT score (Table 1). To investigate putative interaction effects, we ran two full factorial median regressions: TOXO IgG status, diagnostic group status, sex and age on NSE and IL-18. In the NSE analysis, there were no three-way (TOXO status-by-diagnostic group status-by sex, p = 0.442) or two-way interactions (TOXO status-by-diagnostic group status, p = 0.248, TOXO status-by-sex, p = 0.680 or diagnostic group status-by-sex, p = 0.561). Similarly, the corresponding p values for the IL-18 analysis were 0.836, 0.786, 0.721 and 0.945. Our final models were MWU of TOXO status on NSE and IL-18. TOXO+ participants had significantly higher NSE (p < 0.001) (Fig. 1) and IL-18 (p < 0.001) (Fig. 2) levels relative to TOXO− participants. Median NSE in TOXO− and TOXO+ participants were 2200 pg/ml and 3713 pg/ml, respectively, whereas the corresponding statistics for IL-18 were 674 pg/ml and 1068 pg/ml.

### Post-hoc analysis

#### Toxoplasma gondii seropositivity associated with higher levels of neuron-specific enolase and interleukin-18 among both patients and healthy controls

To determine whether TOXO seropositivity was associated with higher NSE and IL-18 levels in patients with SMI and HC separately, we stratified the analysis by diagnostic group (SMI/HC). The bivariate analysis of the patient group (n = 735) showed that TOXO+ and TOXO− patients did not significantly differ in any of the analyzed variables (Table 2). Full factorial median regressions (TOXO IgG status, sex, TOXO IgG status-by-sex, and age) on NSE and IL-18 showed no interaction effects (TOXO IgG status-by-sex, p = 0.382 and p = 0.745 for the NSE and IL-18 analysis, respectively). MWU showed that TOXO+ patients had significantly higher NSE (p < 0.001) (Fig. 1) and IL-18 (p = 0.015) (Fig. 2) levels than TOXO− patients. Median NSE in TOXO− and TOXO+ patients were 1946 pg/ml and 2727 pg/ml, respectively, whereas the corresponding statistics for IL-18 were 748 pg/ml and 1089 pg/ml. In the bivariate analysis among HC (n = 518), TOXO+ and TOXO− HC did not significantly differ in any of the analyzed variables (Table 2). Full factorial median regressions (TOXO IgG status, sex, TOXO IgG status-by-sex, and age) on NSE and IL-18 showed no interaction effects (TOXO status-by-sex, p = 0.379 and p = 0.741 for the NSE and IL-18 analysis, respectively). MWU showed that TOXO+ HC had significantly higher NSE (p < 0.001) (Fig. 1) and IL-18 (p < 0.001) (Fig. 2) levels than TOXO− HC. Median NSE in TOXO− and TOXO+ HC were 2401 pg/ml and 4326 pg/ml, respectively, whereas the corresponding statistics for IL-18 were 606 pg/ml and 1006 pg/ml.

#### Herpesviridae seropositivity not associated with neuron-specific enolase or interleukin-18 levels

To determine TOXO specificity on NSE and IL-18, we investigated the putative associations of CMV IgG status and HSV1 IgG status with NSE and IL-18 following the same methodology as for the TOXO analysis in the whole sample. Bivariate analysis showed that CMV+ participants were 2 years older than CMV− participants, HSV1+ participants were 3 years older and had higher BMI than HSV1− participants, whereas no other differences were detected between seropositive and seronegative participants (Suppl. Table 1 and 2). Full factorial median regressions showed no three-way or two-way interaction effects on neither NSE nor IL-18 (suppl. material). MWU showed that CMV+ (n = 712) and CMV− (n = 541) participants did not differ in NSE (p = 0.794) or IL-18 (p = 0.479) levels, and similarly, HSV1+ (n = 567) and HSV1− (n = 686) participants did not differ in NSE (p = 0.749) or IL-18 (p = 0.273) levels. Due to the age difference between CMV+ and CMV− participants, our final models were median regressions of CMV status on NSE and IL-18 whilst controlling for age. As in MWU, CMV seropositivity was not associated with NSE (p = 0.516) or IL-18 (p = 0.855). Similarly, as HSV1+ participants were older and had higher BMI than HSV1− participants, our final models were median regressions of HSV1 status on NSE and IL-18 whilst controlling for age and BMI. As in MWU, HSV1 seropositivity was neither associated with NSE (p = 0.658) nor IL-18 (p = 0.176).

## Discussion

In the present study, we showed that TOXO+ participants had significantly higher circulating NSE and IL-18 concentrations compared with TOXO– participants. We did not find a SMI specificity as the positive associations between TOXO IgG seropositivity and NSE and IL-18 levels were similar when patients and HC were analyzed separately, with no signs of interaction. Further, NSE and IL-18 levels were correlated in the whole sample, and within patient and HC groups, and in both TOXO− and TOXO+ participants which may suggest an inflammasome-related neuronal damage irrespective of patient/control and TOXO status.

The TOXO-IL-18 association observed in all studied groups combined with the lack of SMI specificity can be indicative of a universal impact of TOXO infections on inflammasome activation with possible implications for the general population. Inflammasome activation has been linked to cognitive dysfunction in neurodegenerative disorders^31^ as well as chronic stress, depression, hypothalamic–pituitary–adrenal (HPA) axis dysfunction and systemic illnesses including cardiovascular diseases, diabetes mellitus and autoimmune diseases^32,33^. Interestingly, HPA dysfunction has been linked to risk for suicide irrespective of the presence of psychiatric symptoms^34^. We can therefore not exclude that the suggested TOXO-related inflammasome activation might increase the susceptibility to psychiatric, systemic illnesses or even suicide. The TOXO-NSE association found in both patients and HC may reflect an ongoing neuronal damage in TOXO-exposed individuals. Indeed, increased NSE levels are thought to reflect the severity of the chronic, ongoing type of neuronal damage and is thus a relevant biomarker for monitoring CNS pathologies, such as infections^35^. The suggested progressive TOXO-related and inflammasome-mediated neuronal damage may have a deleterious impact on the CNS of human hosts.

The association between TOXO infection and IL-18 found in this study has not been previously investigated in human hosts, but is in line with animal studies implicating TOXO in inflammasome activation and production of IL-18^12,36^. In particular, TOXO-infected mice had a substantial increase in IL-18 levels, while IL-18 deficient mice showed enhanced TOXO loads and increased mortality^12^. Further, in TOXO-infected immunodeficient mice, IL-18 administration led to increased interferon-gamma, decreased TOXO loads and decreased mortality^36^. Thus, in experimental models, inflammasome activation following TOXO infection is a critical component of parasite clearance and promotes host survival. However, while increased IL-18 levels could promote adequate immune responses during acute infection, a subtle longstanding inflammasome activation with IL-18 release could have detrimental effects. We have recently demonstrated, in a sample overlapping with the present study, systemic dysregulation of the IL-18 cytokine family in SMI, including elevated IL-18 levels^13^. While our results on IL-18 are suggestive of a TOXO-induced systemic inflammasome activation in both patients and HC, the latent infection could have an additive impact on the already increased IL-18 levels of patients with SMI^13^ which may result in more detrimental consequences in these patients relative to HC.

Even though the TOXO-NSE association has also not been previously explored in human hosts, the increased NSE in TOXO+ participants is in accordance with an experimental study where TOXO-infected mice showed higher brain NSE expression relative to non-infected mice, with the highest increase early after the infection^18^. NSE is not only implicated in neuronal damage, but also in neuronal differentiation, maturation and migration^14–16^. We have recently reported, studying a sample of patients with SMI and HC overlapping with the present study, lower circulating NSE levels in both adults and adolescents with SMI relative to HC, suggestive of a NSE-related neurodevelopmental disturbance^29^. In the present study, patients had also lower NSE than HC, while among all participants as well as within each diagnostic group (patients and HC), TOXO+ participants had higher NSE concentrations than TOXO− participants. Our results may suggest that two different processes affect NSE levels in TOXO+ patients: a neurodevelopmental disturbance related to the disorder leads to lower NSE in this group while TOXO-related neuronal injury increases NSE.

In our post-hoc analyses, we studied two common herpesviruses (CMV and HSV1) which, as with TOXO, are neurotropic pathogens that typically establish life-long latency in human hosts^37–39^. CMV^40–42^ and HSV1^43–45^ are the best studied herpesviruses in relation to cognitive and MRI measures in SMI. In the present study, we did not find any associations between CMV or HSV1 seropositivity and NSE or IL-18, which suggests some specificity of the TOXO infection. The non-elevated NSE and IL-18 levels in CMV and HSV1 seropositive participants relative to seronegative participants may be due to an absence of a substantial ongoing impact of the viruses on the CNS. The previously reported brain structure and cognitive aberrations might thereby be related to the primary viral infection or viral reactivations.

The present study has certain limitations. First, the study is cross-sectional and causality cannot be determined. Although rather unlikely, we cannot exclude a reverse causation, meaning that individuals with higher inflammasome activation might contract TOXO-infections more often than those with lower inflammasome activation. Further, TOXO as well as CMV and HSV1 IgG seropositivity indicates exposure to the pathogens, but the time of the primary infection or the subsequent reactivations cannot be specified. In addition, even though the studied sample was well-characterized, we cannot exclude that the TOXO-IL-18 and the TOXO-NSE associations may have been confounded by unidentified factors that we have not been able to control for. Further, even though NSE is thought to be the main biomarker indicating neuronal damage^17^ we cannot exclude alternative interpretations of the elevated NSE levels in TOXO+ participants. NSE is a glycolytic enzyme^15^ and further, it is present not only in neurons, but also in neuroendocrine cells^14^, with increased circulating levels found in patients with neuroendocrine tumours ^15^. The elevated NSE levels in TOXO+ participants may thereby suggest altered neuronal glycolysis or neuroendocrine system dysregulation. Finally, TOXO infection was associated with inflammasome activation, but the proposed connection to heightened vulnerability to psychiatric and medical conditions are necessarily speculative, as the observed IL-18 increase may lack clinical significance.

To conclude, TOXO seropositivity (which mirrors past infection and current latency) was linked to increased circulating IL-18 and NSE levels, irrespective of diagnostic status. IL-18 and NSE were correlated irrespective of diagnostic and TOXO status. The findings suggest novel relationships between TOXO and IL-18 and NSE, and given that these relationships are not different between patients with SMI and HC, the findings may reflect basic immunological mechanisms and neuronal damage not related to SMI. The elevated TOXO-related NSE can reflect progressive neuronal damage while the increased inflammasome activation might render TOXO-exposed individuals susceptible to both psychiatric and systemic diseases. Further scientific investigation is warranted to establish the connection between TOXO infections and such chronic illnesses, as well as to investigate whether exposed individuals might benefit from antiprotozoal medication.

### Supplementary Information

Supplementary Information.

## Data Availability

Data supporting the findings of the present study have repository at NORMENT/Oslo University Hospital. Restrictions apply to the availability of data and are thereby not publicly available. Data can be made available under reasonable request to the corresponding author and with permission of NORMENT/Oslo University Hospital, in accordance with the ethics agreements/research participants consent.
