# Supplementary material for: Toxoplasma gondii infection associated with inflammasome activation and neuronal injury
Source: Sci Rep. 2024 Mar 4;14:5327. doi: 10.1038/s41598-024-55887-9 (PMC10912117; doi:10.1038/s41598-024-55887-9)
Supplement: Supplementary file 1 — Supplementary Information. [file 41598_2024_55887_MOESM1_ESM.pdf]

Supplementary material

Toxoplasma gondii infection associated with inflammasome activation and neuronal injury

Dimitrios Andreou, Nils Eiel Steen, Lynn Mørch-Johnsen, Kjetil Nordbø Jørgensen, Laura A. Wortinger, Claudia Barth, Attila Szabo, Kevin S. O'Connell, Tove Lekva, Gabriela Hjell, Ingrid Torp Johansen, Monica B. E. G. Ormerod, Unn K. Haukvik, Pål Aukrust, Srdjan Djurovic, Robert H. Yolken, Ole A. Andreassen, Thor Ueland, Ingrid Agartz

Distribution of neuron-specific enolase and interleukin-18

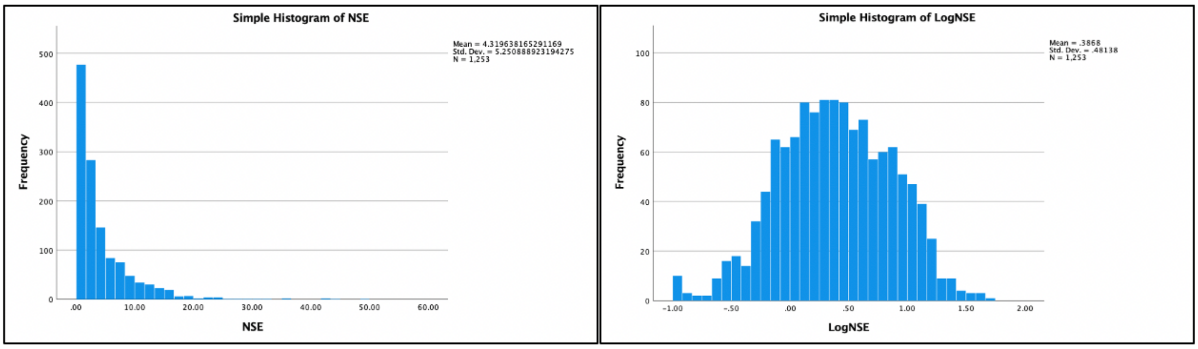

Suppl. Figure 1. Distribution of plasma neuron-specific enolase (NSE) (ng/ml) and logNSE (logarithmic transformation; $\log_{10}$) concentrations in the whole sample (n=1253). The distribution of logNSE was approximately normally distributed as evaluated by visual inspection

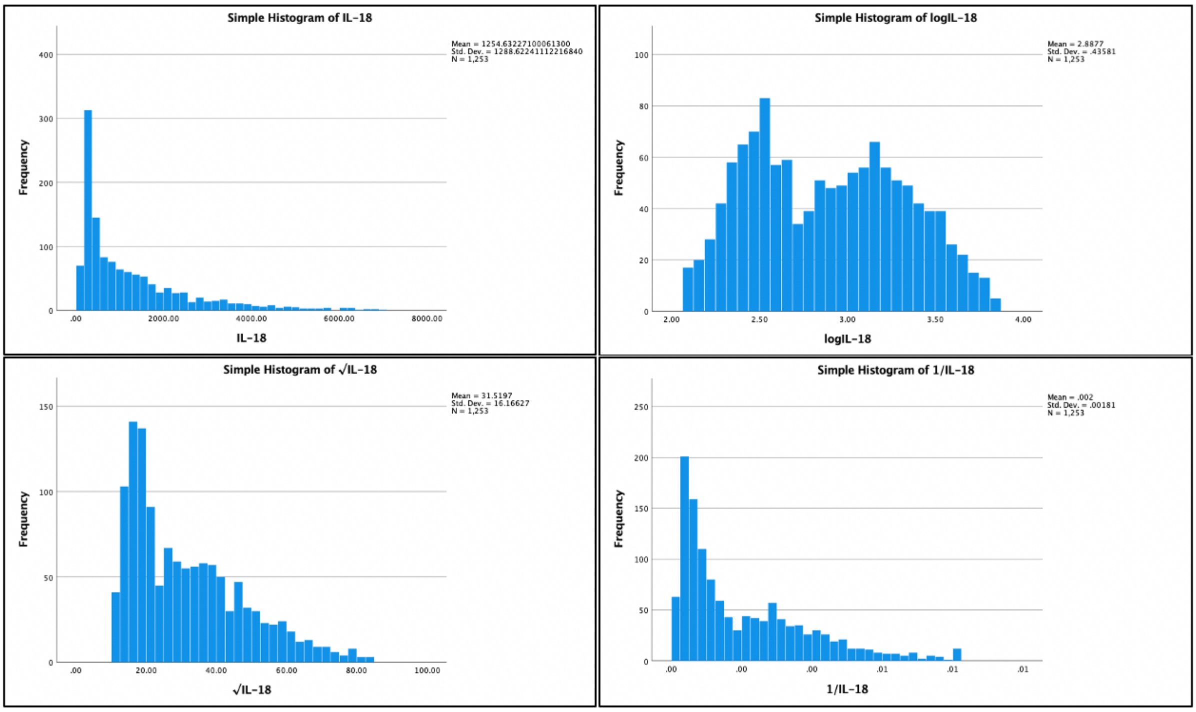

Suppl. Figure 2. Distribution of plasma interleukin-18 (IL-18) (pg/ml), logIL-18 (logarithmic transformation; $\log_{10}$), √IL-18 (square root transformation) and 1/IL-18 (reciprocal transformation) concentrations in the whole sample (n=1253)

Cytomegalovirus analysis

	CMV-		CMV+		
	N[1]	Mean (SD) or %	N[1]	Mean (SD) or %	P value[2]
Patient/control status (% patients)	541	56.4	712	60.4	0.153
Sex (% females)	541	43.1	712	47.8	0.099
Age (years)	541	31.4 (9.7)	712	33.2 (10.3)	**0.002**
Education years	513	13.3 (2.4)	670	13.3 (2.6)	0.941
Handedness (right-handedness %)	513	86.9	669	90.4	0.058
BMI (kg/m^2)	509	25.4 (4.6)	664	25.8 (4.7)	0.101
AUDIT	363	7 (5.4)	476	6.6 (6.1)	0.299
DUDIT	378	2.3 (5.7)	500	2.2. (6.1)	0.897

Suppl. table 1. Group differences between cytomegalovirus (CMV) immunoglobulin G (IgG) seronegative (CMV-) and seropositive (CMV+) participants in patient/control status, sex, age, education years, handedness (right-handedness vs. left-handedness/ambidexterity), body mass index (BMI), alcohol use disorder identification test (AUDIT) score and drug use disorder identification test (DUDIT) score in the whole sample. P-values <0.05 shown in bold

[1]Number of participants with data for each variable
[2]Chi-square test or t-test

To investigate putative interaction effects, we ran full factorial median regressions: cytomegalovirus (CMV) status, diagnostic group status, sex and age on NSE and IL-18. In the NSE analysis, there were no three-way (CMV status-by-diagnostic group status-by sex, p=0.724) or two-way interactions (CMV status-by-diagnostic group status, p=0.845, CMV status-by-sex, p=0.419 or diagnostic group status-by-sex, p=0.642). The corresponding p-values for the IL-18 analysis were 0.488, 0.840, 0.776 and 0.764.

Herpes simplex virus 1 analysis

	HSV1-		HSV1+		
	N[1]	Mean (SD) or %	N[1]	Mean (SD) or %	P value[2]
Patient/control status (% patients)	686	57.7	567	59.8	0.461
Sex (% females)	686	46.6	567	44.6	0.474
Age (years)	686	31.2 (9.9)	567	33.8 (10.1)	**<0.001**
Education years	650	13.4 (2.6)	553	13.3 (2.5)	0.347
Handedness (right-handedness %)	649	88.6	533	89.3	0.700
BMI (kg/m^2)	646	25.4 (4.6)	527	25.9 (4.7)	**0.049**
AUDIT	461	6.7 (5.5)	378	6.9 (6.2)	0.621
DUDIT	472	2.1 (5.5)	406	2.5 (6.4)	0.310

Suppl. Table 2. Group differences between herpes simplex virus 1 immunoglobulin G (IgG) seronegative (HSV1-) and seropositive (HSV1+) participants in patient/control status, sex, age, education years, handedness (right-handedness vs. left-handedness/ambidexterity), body mass index (BMI), alcohol use disorder identification test (AUDIT) score and drug use disorder identification test (DUDIT) score in the whole sample. P-values <0.05 shown in bold

[1]Number of participants with data for each variable
[2]Chi-square test or t-test

To investigate putative interaction effects, we ran full factorial median regressions: herpes virus simplex 1 (HSV1) status, diagnostic group status, sex, age and BMI on NSE and IL-18. In the NSE analysis, there were no three-way (HSV1 status-by-diagnostic group status-by sex, p=0.889) or two-way interactions (HSV1 status-by-diagnostic group status, p=0.364, HSV1 status-by-sex, p=0.479 or diagnostic group status-by-sex, p=0.229). The corresponding p-values for the IL-18 analysis were 0.372, 0.203, 0.375 and 0.336.
